# Aripiprazole as protector against COVID-19 mortality

**DOI:** 10.1038/s41598-024-60297-y

**Published:** 2024-05-29

**Authors:** C. Loucera-Muñecas, M. Canal-Rivero, M. Ruiz-Veguilla, R. Carmona, G. Bostelmann, N. Garrido-Torres, J. Dopazo, B. Crespo-Facorro

**Affiliations:** 1 Computational Medicine Platform, Andalusian Public Foundation Progress and Health-FPS, Seville, Spain; 2 Computational Systems Medicine, Institute of Biomedicine of Seville (IBiS), University Hospital Virgen del Rocío, Consejo Superior de Investigaciones Científicas, University of Seville, Seville, Spain; 3 Hospital Universitario Virgen del Rocío, Av. Manuel Siurot, s/n, 41013 Sevilla, Spain; 4 Centro Investigación Biomédica en Red Salud Mental, CIBERSAM, Madrid, Spain; 5 Instituto de Biomedicina de Sevilla (IBiS), HUVR/CSIC/Universidad de Sevilla, Seville, Spain; 6 Department of Psychiatry, Universidad de Sevilla, Seville, Spain; 7 Centro de Investigación Biomédica en Red de Enfermedades Raras (CIBERER), Seville, Spain; 8 FPS, ELIXIR-Es, Hospital Virgen del Rocío, Seville, Spain

**Keywords:** COVID-19, Antipsychotics, Mortality, SARS-CoV-2, Medical research, Risk factors

## Abstract

The relation of antipsychotics with severe Coronavirus Disease 19 (COVID-19) outcomes is a matter of debate since the beginning of the pandemic. To date, controversial results have been published on this issue. We aimed to prove whether antipsychotics might exert adverse or protective effects against fatal outcomes derived from COVID-19. A population-based retrospective cohort study (January 2020 to November 2020) comprising inpatients (15,968 patients) who were at least 18 years old and had a laboratory-confirmed COVID-19 infection. Two sub-cohorts were delineated, comprising a total of 2536 inpatients: individuals who either had no prescription medication or were prescribed an antipsychotic within the 15 days preceding hospitalization. We conducted survival and odds ratio analyses to assess the association between antipsychotic use and mortality, reporting both unadjusted and covariate-adjusted results. We computed the average treatment effects, using the untreated group as the reference, and the average treatment effect on the treated, focusing solely on the antipsychotic-treated population. Among the eight antipsychotics found to be in use, only aripiprazole showed a significant decrease in the risk of death from COVID-19 [adjusted odds ratio (OR) = 0.86; 95% CI, 0.79–0.93, multiple-testing adjusted p-value < 0.05]. Importantly, these findings were consistent for both covariate-adjusted and unadjusted analyses. Aripiprazole has been shown to have a differentiated beneficial effect in protecting against fatal clinical outcome in COVID-19 infected individuals. We speculate that the differential effect of aripiprazole on controlling immunological pathways and inducible inflammatory enzymes, that are critical in COVID19 illness, may be associated with our findings herein.

## Introduction

The outbreak originated by Severe Acute Respiratory Syndrome due to Coronavirus-2 (SARS-CoV-2) started at the end of 2019 in Wuhan, China. The World Health Organization declared it a global pandemic in March 2020^1^. At the time this article was written, a new SARS-CoV-2 variant concerns the scientific and health authorities^2^. As a result, it has been observed an increase in infections and hospitalizations due to COVID-19 across worldwide^3–5^ and some World Health Organization (WHO) regions such as Western Pacific Regions have experienced an increase in the number of deaths^6^. The new COVID-19 waves as well as the potential mutations of the virus represent a new risk for the general population and particularly for those at high risk^7^.

It has been suggested that the psychosocial effects of SARS-CoV-2 on the population will lead to an increase in the use of medication in treating mental health conditions, thus the potential effects of these medications should be considered in the light of COVID-19 treatment^8^. A risen in the prescription of antipsychotics in at-risk populations after the pandemic caused by COVID-19 has been recently described^9^.

Controversial results have been published on the potential role of antipsychotics in COVID-19. While some studies pointed out possible adverse effects^10,11^, others have reported protective effects against infection and prognosis of the disease caused by SARS-CoV-2^12,13^. During SARS-CoV-2 infection multiple inflammatory pathways are activated that promotes secretion of proinflammatory cytokines^14^. Antipsychotics exert anti-inflammatory effects via reducing proinflammatory cytokines production, modulating monocytes response through Toll-Like Receptors (TLR) and inhibiting microglial activation^15–17^. In humans, the immunomodulatory effect of risperidone and aripiprazole has been demonstrated^18^, with aripiprazole demonstrating a greater anti-inflammatory effect on TNF-α, IL-13, IL-17α and fractalkine. Nevertheless, important limitations have been highlighted in these studies such as the need to explore specific types of antipsychotics, doses, or the inclusion of relevant covariables in the analyses^19^. In fact, it has been emphasized the necessity of combining experimental and clinical studies to elucidate the relevance and repercussions that could have the use of antipsychotics during COVID-19 era^20^.

The aim of this study was to investigate if antipsychotic treatments have a protective or adverse impact on COVID-19 related deaths. We leveraged the Andalusian Public Health System's Health Population Base (BPS), a vast electronic health record (EHR) resource established in 2001. Encompassing over 13 million users, the BPS represents one of the world's most extensive clinical data repositories^21^. The BPS's size and comprehensiveness create an exceptional environment for conducting large-scale real-world evidence (RWE) studies. Based on our previous studies^22,23^ we hypothesized that persons under aripiprazole treatment will show reductions in the risk of death by COVID-19.

## Materials

### Study design

The study was approved by the Ethics Committee for the Coordination of Biomedical Research in Andalusia (29 September 2020, Acta 09/20) and by the Local Ethics Committee of Virgen del Rocío University Hospital (PI-2578-N-20). Informed consent was not required for the secondary use of clinical data for research. For secure real-world data analysis, the study utilized the Infrastructure for secure generation of Real-World Evidence from Real World Data from the Andalusian Health Population Database (iRWD) managed by the Foundation for Progress and Health of the Andalusian Public Health System. The data was securely transferred from the BPS to the iRWD, thereby ensuring the safety and integrity of the information throughout the process.

The study consists of a retrospective cohort of Andalusian patients hospitalized with a COVID-19 diagnosis from January 2020 to November 2020, as registered in the BPS. The sample (n = 2.536) consisted of inpatients who met the following criteria: age of 18 years or older, having a laboratory-confirmed COVID-19 infection (either PCR or antigen test) and who were either not prescribed any medication or were prescribed an antipsychotic during the 15 days prior to the hospitalization event. We identified 15,968 inpatients who met the inclusion criteria before selecting the antipsychotic-treated and untreated subcohort.

### Covariate, event, and endpoint definition

To reduce the high dimensionality induced by the ICD codes we grouped the conditions as: obesity and other associated conditions (E66), diabetes mellitus (E11), circulatory (I00–I99), respiratory (J00–J99), neoplasms (C00–D49), dementia (F00–F03), anxiety or mood disorders (F30–F48), and other mental diseases (F04–F29 and F50–F99). In addition, we obtained the following data for each patient: sex, age categorized as [18, 41), [41, 68) and [68, 99), flu vaccination status, and pneumococcal vaccination status. This categorization follows^24^ and^25^. Finally, we used death from COVID-19 as the primary outcome, defined as the endpoint by a certified death event during the first 30 days of a COVID-19 hospitalization, based on^26^.

### Treatment definition

The seven antipsychotics examined in this analysis are Quetiapine, Sulpiride, Paliperidone, Aripiprazole, Haloperidol, Olanzapine, and Risperidone (all the antipsychotics found in the population under study). A patient was considered to be under a specific treatment if they received a dispensation of that treatment between 15 days prior to the index date and the index date itself. In addition to antipsychotic medications, we investigated whether each patient had received any anticoagulant medications during the same timeframe (between 15 days before and the index date). To reduce the dimensionality introduced by the variety of anti-inflammatory treatments used by the cohort, we categorized patients into two groups: those receiving any anti-inflammatory medication and those not receiving any (See Supplementary Material).

### General description of the population under study

Table 1 shows the characteristics of the cohort studied. The cohort is divided into those who died and those who survived. The table reveals several key differences between the two groups. Notably, the deceased group had a significantly higher average age (79.09 years) compared to survivors (62.89 years). Vaccination rates were also higher among deceased patients, with a greater percentage having received flu and pneumococcal vaccinations. Perhaps most significantly, the table suggests a higher prevalence of underlying medical conditions in the deceased group, including diabetes, circulatory diseases, neoplasms, respiratory diseases, and dementia. Interestingly, the data also shows that Quetiapine (DB01224), an antipsychotic medication, was used more frequently by deceased patients (22.4%) compared to survivors (15.1%), whereas out of the 31 patients that were using Aripiprazole only 1 died, highlighting a potential difference in the impact of these medications on mortality risk. As expected, Table 1 shows a cohort where deceased patients were older, had a higher prevalence of vaccinations and underlying health conditions, and were more likely to have used Quetiapine.

**Table 1:** Covariate associations with end point: Chi-squared tests, p-values, counts, and proportions.

	Death (no)	Death (yes)	p-value
N	1968	568	
Sex (female) (%)	896 (45.5)	249 (43.8)	0.506
Flu vaccine (%)	756 (38.4)	336 (59.2)	< 0.001
Pneumococcal vaccine (%)	438 (22.3)	196 (34.5)	< 0.001
Obesity (%)	249 (12.7)	80 (14.1)	0.410
Diabetes (%)	461 (23.4)	191 (33.6)	< 0.001
Circulatory (%)	991 (50.4)	416 (73.2)	< 0.001
Neoplasms (%)	197 (10.0)	85 (15.0)	0.001
Respiratory (%)	323 (16.4)	125 (22.0)	0.003
Dependences (%)	139 (7.1)	30 (5.3)	0.160
Dementia (%)	338 (17.2)	188 (33.1)	< 0.001
Anxiety or mood disorders (%)	395 (20.1)	113 (19.9)	0.973
Other mental diseases (%)	644 (32.7)	192 (33.8)	0.666
Age [mean (SD)]	62.89 (18.64)	79.09 (11.94)	< 0.001
Other antiinflammatory (%)	1779 (90.4)	507 (89.3)	0.472
Sulpiride (DB00391) (%)	103 (5.2)	29 (5.1)	0.989
Quetiapine (DB01224) (%)	297 (15.1)	127 (22.4)	< 0.001
Paliperidone (DB01267) (%)	27 (1.4)	9 (1.6)	0.860
Aripiprazole (DB01238) (%)	30 (1.5)	1 (0.2)	0.018
Haloperidol (DB00502) (%)	155 (7.9)	51 (9.0)	0.447
Olanzapine (DB00334) (%)	63 (3.2)	17 (3.0)	0.909
Risperidone (DB00734) (%)	104 (5.3)	42 (7.4)	0.072

### Drug processing

Drug prescription data of Andalusian public health care patients were received in three separate data dumps from the BPS database: the first one on Nov 17, 2020 with 12,009 prescription entries containing any one of the seven antipsychotics, and the second and third ones on Feb 15, 2021 with 13,413 and 19,391 entries, respectively. The data sets were combined into one and parsed as follows: each drug prescription entry was broken down into its active constituent agents, and for each patient, each of their prescription date and each active agent the following fields were extracted:

- Encrypted NUHSA code (patient identifier)
- Description of the prescribed medication
- Active agent
- Amount of agent per unit (e.g. per capsule, tablet, solution, or injection)
- Dose/mode of administration
- The total amount administered by prescription
- Prescription date

In the case of the seven antipsychotics at hand, there is only one active ingredient per prescription, which is the antipsychotic itself, so the number of entries/rows before and after parsing remains the same. Duplicate entries, which might have resulted from overlaps in the data dumps, were removed. The resulting data set contained 33,348 entries.

Next, for each entry the number of days between the prescription date and that of the subsequent entry (same patient, active agent), if there was any, was calculated. Based on this number of days between two given subscriptions and the total amount of active agents administered in the preceding prescription, which had previously been determined, the average daily dose of the preceding prescription was calculated. This was done for all prescriptions followed by a subsequent prescription. Finally, for each patient and each active ingredient occurring in their prescriptions, an average over all the average daily doses was calculated. So, the following three fields were added to the data set:

- Days to next prescription
- The average daily dose of prescription
- Total average of all average doses (patient-wise, per active agent)

Drug information is summarized in Table 2.

**Table 2:** Antipsychotic type, mode of administration and dosage.

Mode of administration	# Patients	Mean # prescriptions per patient	Mean daily dose^a^	Mean time span between prescriptions			NA's^b^
				Min	Median	Max	
Aripiprazole							
1 IM injection at 300 mg	3	17.7	19.7 mg/day	17d	27d	31d	0
1 IM injection at 400 mg	5	12.4	14 mg/day	28d	29d	32d	0
150 ml oral solution at 1 mg/ml	2	11.5	22.5 mg/day	16d	22d	28d	0
28 tablets at 5 mg	11	10.3	5.7 mg/day	21d	27d	82d	2
28 tablets at 10 mg	16	8.3	10 mg/day	23d	32d	54d	3
28 tablets at 15 mg	7	5.6	15.4 mg/day	23d	27d	30d	3
28 tablets at 20 mg	2	10	20.7 mg/day	28d	28d	28d	0
Haloperidol							
5 IM injections at 5 mg	11	1.3	0.2 mg/day	125d	340d	554d	9
15 ml oral solution/dayrops at 2 mg/ml	72	5.5	2.6 mg/day	8d	20d	222d	38
30 ml oral solution/dayrops at 2 mg/ml	217	7.2	4.8 mg/day	5d	36d	561d	75
30 tablets at 10 mg	11	15.7	24.9 mg/day	11d	25d	71d	3
Olanzapine							
1 IM injection at 10 mg	1	2	1 mg/day	10d	10d	10d	0
28 capsules at 2.5 mg	4	7.8	5.1 mg/day	13d	21d	36d	1
28 capsules at 5 mg	7	12	11.5 mg/day	9d	32d	242d	0
28 capsules at 10 mg	5	11.6	15.8 mg/day	10d	25d	31d	1
56 capsules at 10 mg	7	2.3	8.5 mg/day	53d	56d	136d	2
28 capsules at 15 mg	6	18.5	23.1 mg/day	14d	28d	33d	0
28 capsules at 20 mg	3	16	20.4 mg/day	28d	28d	28d	0
28 tablets at 2.5 mg	15	12.7	3.1 mg/day	14d	29d	96d	4
28 tablets at 5 mg	22	21.9	9.7 mg/day	9d	28d	51d	3
28 tablets at 7.5 mg	2	6	13.4 mg/day	13d	20d	26d	0
56 tablets at 7.5 mg	3	4.7	7.5 mg/day	56d	56d	56d	2
28 tablets at 10 mg	13	14.1	16.1 mg/day	9d	23d	51d	3
56 tablets at 10 mg	21	11.7	19.4 mg/day	12d	53d	161d	2
28 tablets at 15 mg	4	3.5	16.6 mg/day	20d	27d	28d	1
28 tablets at 20 mg	3	18.3	20.6 mg/day	28d	28d	28d	0
Paliperidone							
1 prefilled syringe at 75 mg	2	12	2.8 mg/day	29d	29d	29d	1
1 prefilled syringe at 100 mg	6	14.7	3.4 mg/day	28d	30d	37d	0
1 prefilled syringe at 150 mg	10	13.6	5.8 mg/day	20d	28d	52d	0
1 prefilled syringe at 350 mg	2	3.5	3.8 mg/day	92d	92d	92d	0
1 prefilled syringe at 525 mg	1	8	5.6 mg/day	97d	97d	97d	0
28 tablets at 3 mg	11	16.1	4.8 mg/day	14d	28d	44d	0
28 tablets at 6 mg	5	16	7.2 mg/day	13d	30d	50d	0
28 tablets at 9 mg	4	24.5	17.3 mg/day	14d	21d	28d	0
Quetiapine							
6 tablets at 25 mg	6	1.3	14.4 mg/day	6d	23d	40d	4
60 tablets at 25 mg	296	14.3	82.7 mg/day	11d	29d	408d	30
10 tablets at 50 mg	3	21.3	90.4 mg/day	8d	20d	33d	1
60 tablets at 50 mg	137	11.8	157.3 mg/day	11d	31d	174d	23
60 tablets at 100 mg	64	13.7	328.4 mg/day	5d	35d	95d	3
60 tablets at 150 mg	17	9.9	302.5 mg/day	21d	39d	453d	4
60 tablets at 200 mg	27	12.7	643.7 mg/day	10d	55d	68d	4
60 tablets at 300 mg	23	14	854.3 mg/day	15d	32d	126d	3
60 tablets at 400 mg	14	8.4	643 mg/day	22d	60d	66d	3
Risperidone							
1 IM injection at 25 mg	2	28	2 mg/day	14d	15d	17d	0
1 IM injection at 37.5 mg	3	20.3	3 mg/day	14d	15d	16d	0
1 IM injection at 50 mg	3	20.7	3.5 mg/day	14d	14d	25d	0
30 ml oral solution at 5 mg/5 ml	21	10.6	2.7 mg/day	6d	18d	76d	7
100 ml oral solution at 5 mg/5 ml	65	10.2	5.3 mg/day	12d	44d	166d	19
20 tablets at 500ug	2	1.5	714.3ug/day	14d	14d	14d	1
28 tablets at 500ug	6	7.2	679.7mcg/day	10d	24d	28d	2
56 tablets at 500ug	15	10.5	1401.7mcg/day	17d	31d	210d	3
60 tablets at 500ug	8	4.8	1593.6mcg/day	19d	30d	224d	2
20 tablets at 1 mg	14	20.9	2.3 mg/day	7d	17d	77d	2
28 tablets at 1 mg	5	5	1 mg/day	27d	28d	29d	3
56 tablets at 1 mg	6	20.3	4.9 mg/day	9d	25d	56d	1
60 tablets at 1 mg	37	10.2	2.4 mg/day	15d	32d	71d	10
28 tablets at 2 mg	1	4	2.3 mg/day	25d	25d	25d	0
56 tablets at 2 mg	1	8	2 mg/day	56d	56d	56d	0
60 tablets at 2 mg	5	19.8	8.9 mg/day	16d	19d	53d	0
20 tablets at 3 mg	2	19.5	8.4 mg/day	10d	23d	36d	0
60 tablets at 3 mg	14	13	7.4 mg/day	20d	36d	62d	3
30 tablets at 6 mg	1	22	5.8 mg/day	33d	33d	33d	0
60 tablets at 6 mg	5	5.8	6 mg/day	56d	60d	95d	0
Sulpiride							
12 injections at 100 mg	2	1	NA	NA	NA	NA	2
30 capsules at 50 mg	153	7.7	86.8 mg/day	4d	30d	519d	73
36 tablets at 200 mg	10	2.9	199.5 mg/day	36d	36d	36d	9

^a^A given patient may be prescribed more than one mode of administration, so the mean daily dose of the patient includes all modes combined.

^b^NA means a given patient only had one prescription of the drug, hence the mean daily dose and the time span between prescriptions cannot be determined.

## Methods

The study procedures were in accordance with local legislation and the Declaration of Helsinki^27^. This study followed the Strengthening the Reporting of Observational Studies in Epidemiology (STROBE) reporting guideline for cohort studies^28^.

To elucidate if any given treatment could potentially reduce the mortality in COVID-19 inpatients we conducted three statistical tests that consider covariates that present an a priori possibility of confounding the association between a treatment and the odds of surviving COVID-19 disease (sex, age, co-morbidities, anti-inflammatory medication use, and influenza and pneumococcal vaccination status):

- The odds outcome was estimated using a general binomial linear model weighted using the inverse probability of treatment weighting (IPTW) technique, with the weights computed by means of a logistic regression model and adjusted for estimating the average treatment effect on the treated population (ATT), conditioned to the confounders of interest using the whole cohort, using the untreated population as the reference.
- In addition, we conducted a similar analysis using the untreated group as the reference. In this case, the covariate adjustment was carried out by estimating the average treatment effect on the population (ATE).
- Finally, we obtained the Kaplan–Meier survival curves for each treatment comparing the untreated versus the treated groups. We provide unadjusted curves along with IPTW covariate-adjusted matched survival curves.

In both cases, we provide FDR-adjusted p values. Significance is achieved at level 0.05, and we provide 95% confidence intervals. To get an accurate measure of the variability of the marginal odds ratio we used heteroskedasticity-consistent standard errors^29^.

The results have been summarized in Table 3.

**Table 3:** Covariate-adjusted Odds Ratio Estimates, Confidence Intervals and FDR-adjusted p-values for the antipsychotics tested.

name	n	OR	OR-CI 5%	OR-CI 95%	p val	p val (fdr)	estimand
Aripiprazole	31	0.865	0.799	0.936	< 0.001	0.002	ATT
		0.796	0.722	0.878	< 0.001	< 0.001	ATE
Haloperidol	206	0.967	0.903	1.036	0.338	0.788	ATT
		0.959	0.895	1.027	0.226	0.620	ATE
Quetiapine	424	1.013	0.948	1.083	0.700	0.788	ATT
		1.017	0.957	1.080	0.596	0.834	ATE
Olanzapine	80	1.014	0.918	1.120	0.788	0.788	ATT
		0.933	0.819	1.063	0.296	0.620	ATE
Risperidone	146	1.020	0.941	1.106	0.627	0.788	ATT
		1.041	0.956	1.133	0.354	0.620	ATE
Paliperidone	36	1.039	0.893	1.210	0.620	0.788	ATT
		0.983	0.714	1.354	0.918	0.985	ATE
Sulpiride	132	0.947	0.865	1.038	0.246	0.788	ATT
		0.998	0.832	1.198	0.985	0.985	ATE

### Software

To perform the analysis, we used:

- R version 3.6.3 (2020-02-29)
- Survival analysis: Survival R package (v 3.2.11)^30^
- Weights computation for IPW: WeightIt R package (v 0.12)^31^
- General linear models: glm R stats core package^32^.
- Survival plots: survminer R package (v 0.4.9)^33^.
- Table 1: tableone R package (v 0.13.2)^34^.
- HC standard errors computation: Sandwich R package (v 3.0.1)^35^

### Ethical standards

The study procedures were in accordance with local legislation and the Declaration of Helsinki. This study followed the Strengthening the Reporting of Observational Studies in Epidemiology (STROBE) reporting guideline for cohort studies. The protocol was approved by the Local Ethics Committee of Virgen del Rocío University Hospital (PI-2578-N-20). All participants data were anonymized to ensure confidentiality.

## Results

**Figure 1:**
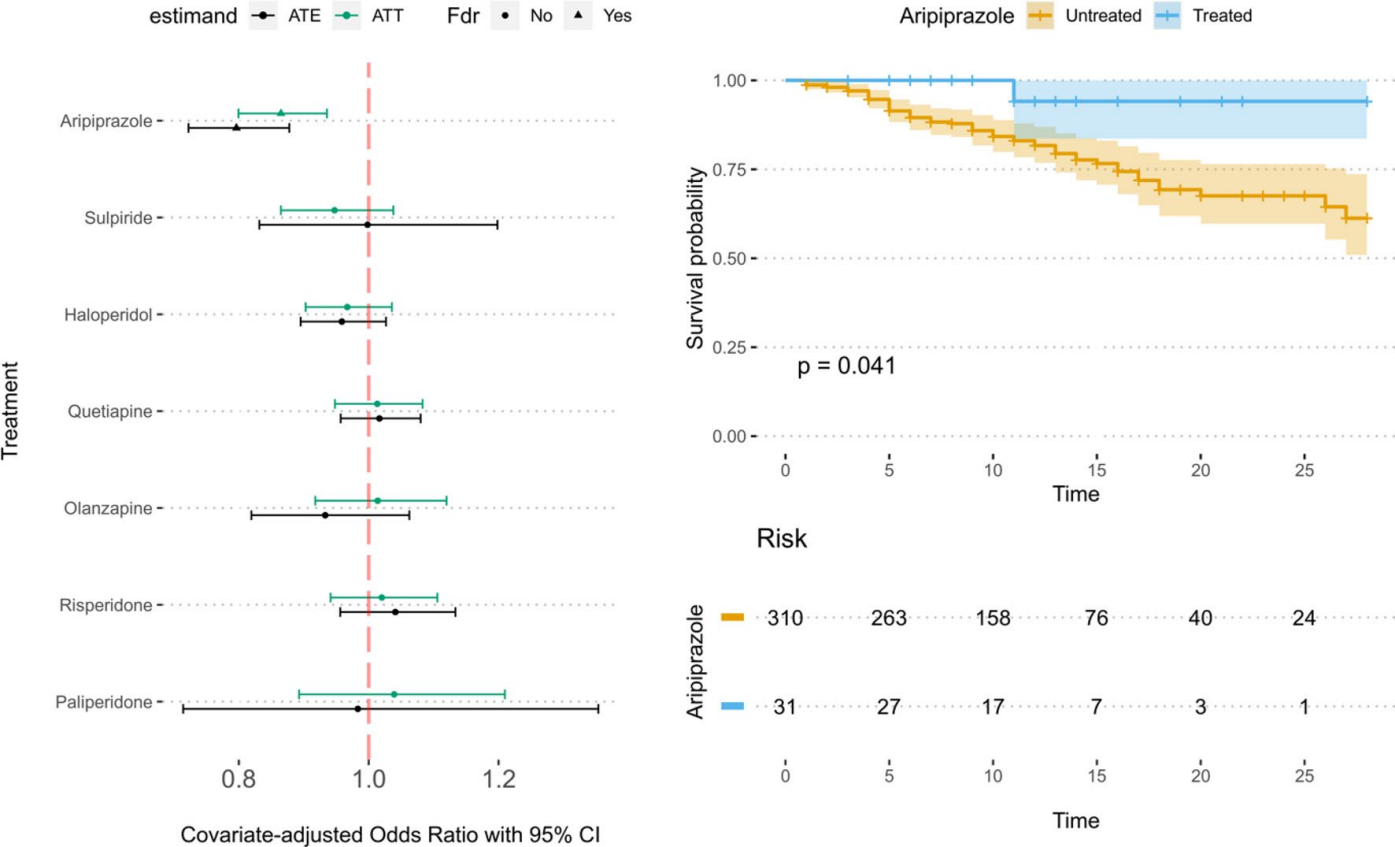
Antipsychotic's impact on patient survival. Left: adjusted odds ratios (with 95% confidence intervals) for all treatments before and after FDR correction, to assess the effect on death after hospitalization. OR below 1 indicate protective effects. Right: Kaplan–Meier survival curves for patients who received Aripiprazole compared to those who didn't, controlling for covariates.

Aripiprazole is the only antipsychotic treatment that shows a protective effect, as can be evidenced by the 95% confidence intervals for the covariate-adjusted odds-ratio analysis (left side figure). Find a survival curve plot for each treatment in Supplementary material. This finding is further reinforced by the covariate-adjusted survival analysis on the matched population (right side figure). See Fig. 1.

## Discussion

The main finding derived from this study is that aripiprazole leads to reduction in the risk of death caused by COVID-19. On the contrary, the rest of the antipsychotic treatments included in the analyses did not significantly reduce the mortality related to COVID-19.

Antipsychotics have been shown to exert anti-inflammatory effects through decreased proinflammatory cytokine production, modulation of monocyte response through TLR and inhibition of microglial activation^16,17^. In fact, Chlorpromazine protects mice from severe clinical disease and SARS-CoV-2^36^. Clozapine an atypical antipsychotic, has been revealed to be effective in suppressing proinflammatory cytokine expression by limiting NLRP3 inflammasome activation in an in vitro model of schizophrenia^37^.

Our group showed that aripiprazole (marked Phenylpiperazine) reverts the changes caused by COVID-19 in gene expression which could validate aripiprazole as a treatment for COVID-19^22^. Interestingly, research investigating approximately 12,000 drugs in clinical-stage or Food and Drug Administration (FDA)-approved small molecules to identify candidate drugs to treat COVID-19, reported that Elopiprazole (a never marketed phenylpiperazine antipsychotic drug) was listed among the 21 most potent compounds to inhibit SARS-CoV infection^38^. A possible explanation for the superiority of aripiprazole, in convergence with a recent publication, is that aripiprazole increases the expression of anti-inflammatory markers compared to other antipsychotics. In particular, aripiprazole induced mTORC1 inhibition, which is an important mechanism of action in microglial cells that leads to an anti-inflammatory shift^39^.

Aripiprazole has been included into the called Functional Inhibition of acid sphingomyelinase (FIASMA) medications^40^. Carpinteiro et al. (2020) suggested that acid sphingomyelinase (ASM)/ceramide system plays an important role in the infection of cells with SARS-CoV-2 since its activation by SARS-CoV-2 facilitates viral entry into cells^41^. FIASMA medications would inhibit ASM and reduce the formation of ceramide-enriched membrane platforms, decreasing cell infection with SARS-CoV-2 and subsequent inflammation^42^. Among the antipsychotics included in the present study, aripiprazole was the only considered FIASMA medication. Aripiprazole property to inhibit ASM as well as its anti-inflammatory effects could explain the superiority showed by the molecule against fatal outcome derived from COVID-19.

None of the antipsychotics included in our analysis contribute significantly to the increase in mortality related to COVID-19 in the adjusted analyses. As was noted previously, former publications which have reported adverse effects of antipsychotics against COVID-19 have shown significant limitations such as considering antipsychotics as homogeneous group^14^. In that sense, previous works that have not differentiated between different types of antipsychotics have reported adverse effects on COVID-19^43–45^. However, those studies that have explored potential adverse effects of specific antipsychotics have not reported a worse prognosis of COVID-19^12,46,47^. These results underline the need to study the possible effects of antipsychotics on a one-to-one basis, as different antipsychotics may have very different effects on each other.

Some limitations should be considered when interpreting the results. Firstly, we have used preprocessed electronic records that could not include sensitive (uncodified) information. Secondly, our treatment information is based on dispensation records, so unless patients are receiving an injectable medication, which is administered at the time of purchase, we cannot be confident that the patient is using the drug. However, we consider that most patients adhere to their prescribed treatment, as the majority present a mean time span between dispensations below 40 days. Finally, aripiprazole resulted to be the least prescribed antipsychotic, which limits the generalizability of the results as well as the performance of complementary analyses that could elucidate which dosage of the drug is more likely to play a protective role for the risk of death. Nevertheless, a strength of the study is that, to the best of our knowledge, this is the first study to examine the mortality of COVID-19 linked to specific antipsychotic drugs, employing a sizable study population for that purpose.

In conclusion, our results exploring a population-based case–control cohort pose the potential usefulness of aripiprazole, in COVID-19 infected individuals with psychiatric disorders. Aripiprazole could play an important role in minimizing the fatal outcomes related to COVID-19. The anti-inflammatory properties of aripiprazole against cytokine storm as well as its FIASMA properties may explain its genuine effects in reducing the risk of death associated with COVID-19.

### Supplementary Information

Supplementary Information 1.

Supplementary Information 2.

## Data Availability

Data is available upon formal request to Carlos Loucera-Muñecas.
