# Supplementary material for: Aripiprazole as protector against COVID-19 mortality
Source: Sci Rep. 2024 May 29;14:12362. doi: 10.1038/s41598-024-60297-y (PMC11137032; doi:10.1038/s41598-024-60297-y)
Supplement: Supplementary file 1 — Supplementary Information 1. [file 41598_2024_60297_MOESM1_ESM.docx]

# Supplementary material

**Aripiprazole confusion**

| Treatment dose at index point | Death (No) | Death (Yes) |
| --- | --- | --- |
| n | 30 | 1 |
| Aripiprazole 300MG/Inyectable | 2 (6.7) | 0 (0.0) |
| Aripiprazole 400MG/Inyectable | 3 (10.0) | 0 (0.0) |
| Aripiprazole 10MG 28 Tablets | 7 (23.3) | 0 (0.0) |
| Aripiprazole 15MG 28 Tablets | 3 (10.0) | 0 (0.0) |
| Aripiprazole 20MG 28 Tablets | 1 (3.3) | 0 (0.0) |
| Aripiprazole 5MG 28 Tablets | 5 (16.7) | 0 (0.0) |
| Aripiprazole 400MG/Inyectable + Risperidone 6MG 60 Tablets | 1 (3.3) | 0 (0.0) |
| Aripiprazole 10MG 28 Tablets + Olanzapine 20MG 28 Tablets | 1 (3.3) | 0 (0.0) |
| Aripiprazole 5MG 28 Tablets + Olanzapine 5MG 28 Tablets | 1 (3.3) | 0 (0.0) |
| Aripiprazole 10MG 28 Tablets + Haloperidol 10MG 30 Tablets | 1 (3.3) | 0 (0.0) |
| Aripiprazole 10MG 28 Tablets + Quetiapine 100MG 60 Tablets | 1 (3.3) | 0 (0.0) |
| Aripiprazole 10MG 28 Tablets + Quetiapine 300MG 60 Tablets | 0 (0.0) | 1 (100.0) |
| Aripiprazole 10MG 28 Tablets + Quetiapine 400MG 60 Tablets | 1 (3.3) | 0 (0.0) |
| Aripiprazole 15MG 28 Tablets + Quetiapine 100MG 60 Tablets | 1 (3.3) | 0 (0.0) |
| Aripiprazole 15MG 28 Tablets + Quetiapine 50MG 60 Tablets | 1 (3.3) | 0 (0.0) |
| Aripiprazole 30MG 28 Tablets + Quetiapine 400MG 60 Tablets + Risperidone 6MG 60 Tablets | 1 (3.3) | 0 (0.0) |
