# Supplementary figures and images for: Aripiprazole as protector against COVID-19 mortality
Source: Sci Rep. 2024 May 29;14:12362. doi: 10.1038/s41598-024-60297-y (PMC11137032; doi:10.1038/s41598-024-60297-y)

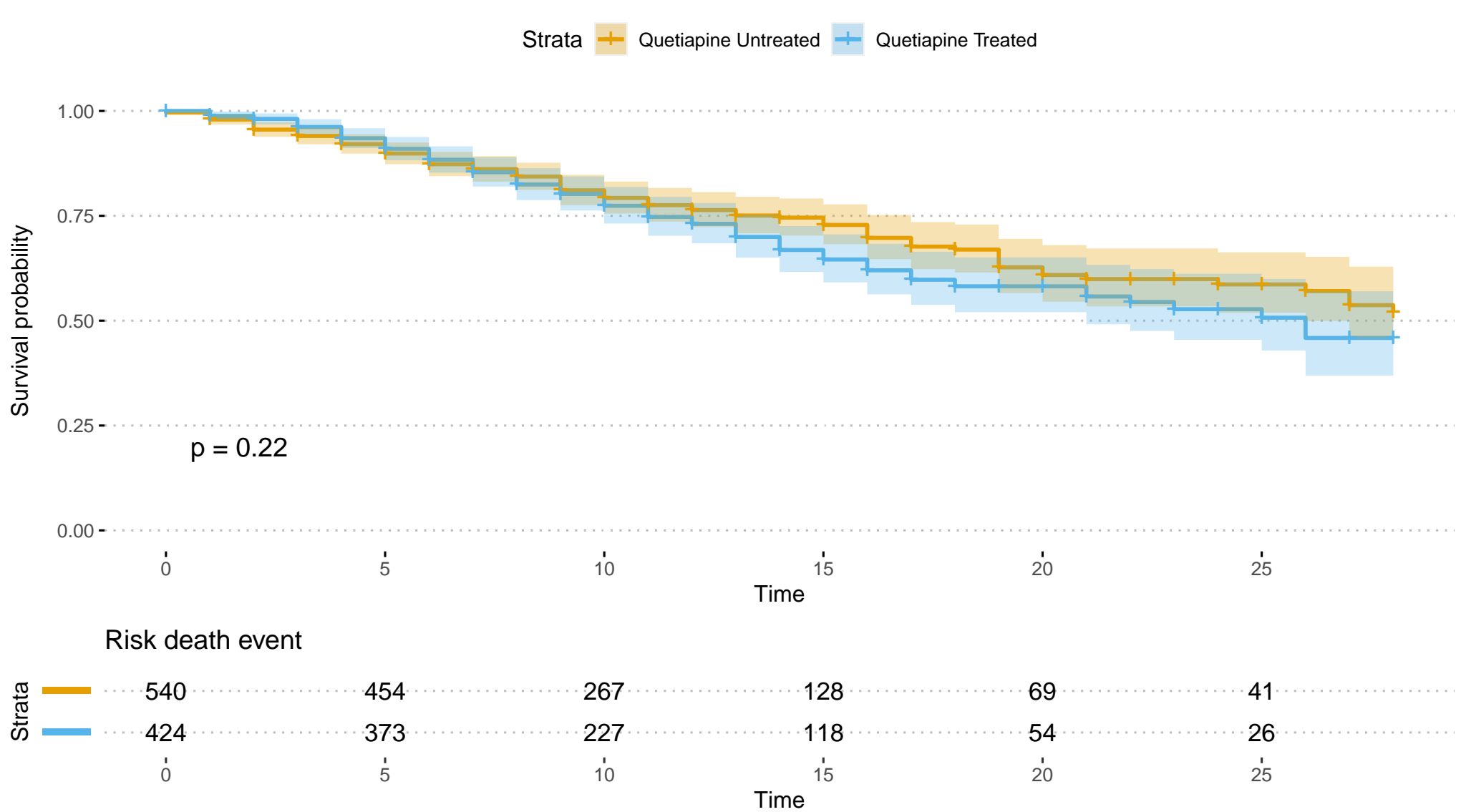

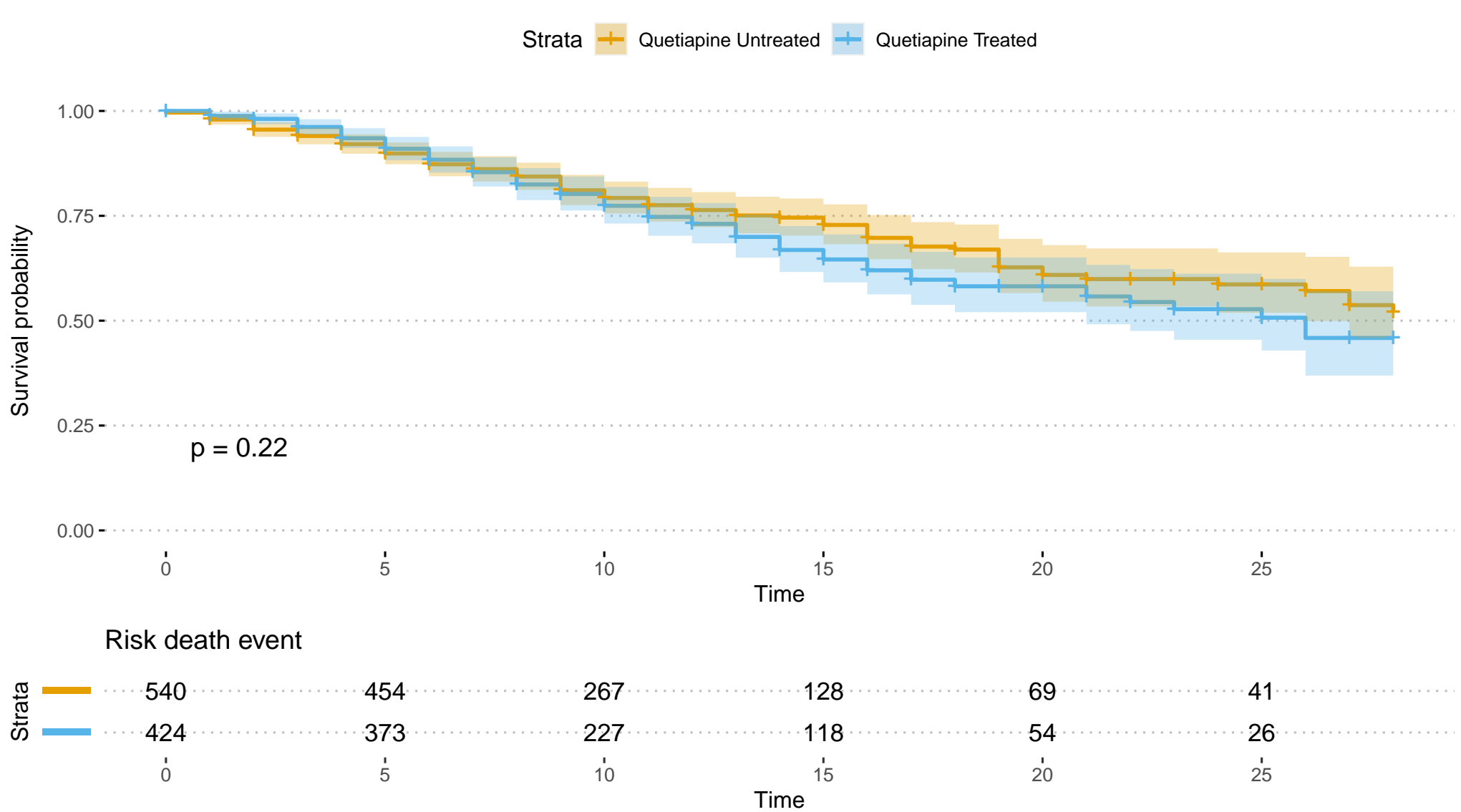

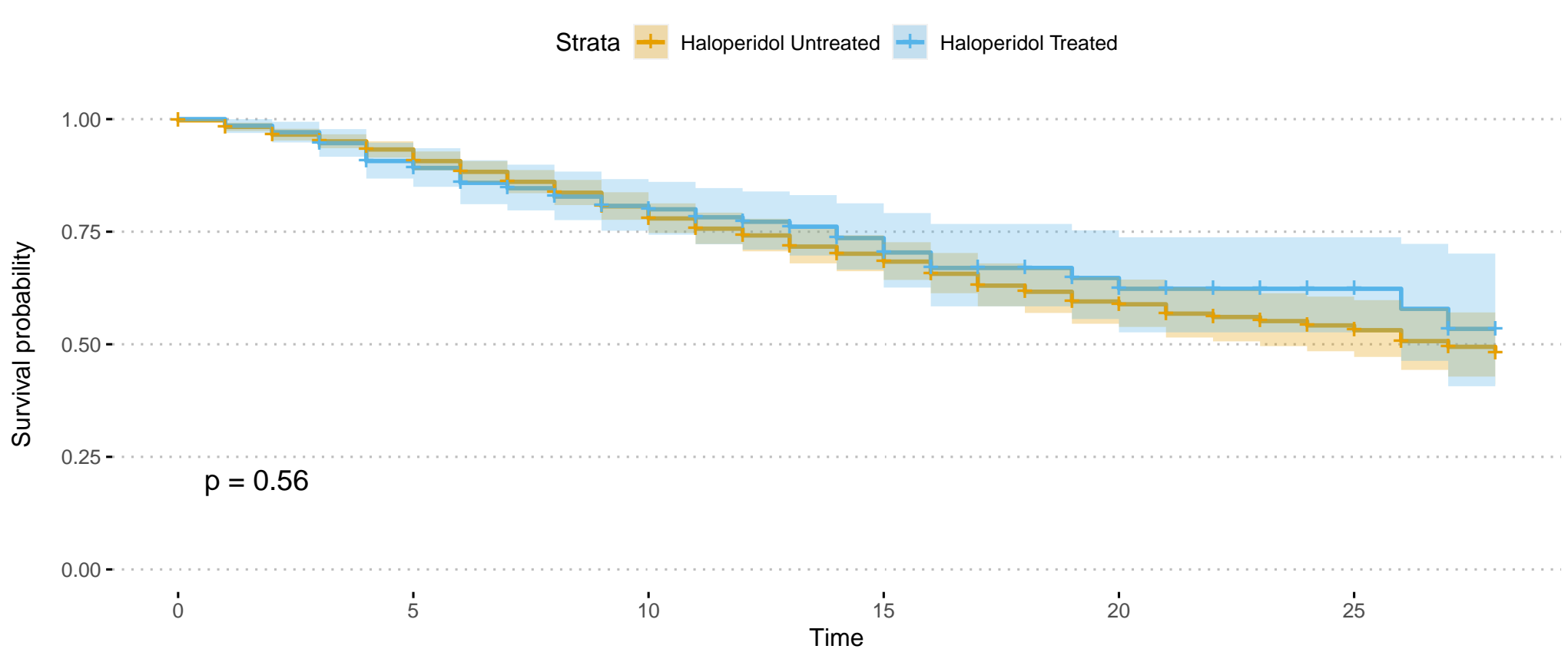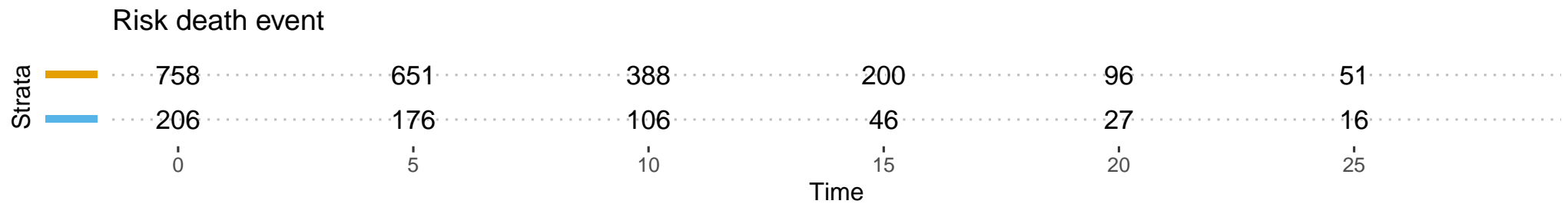

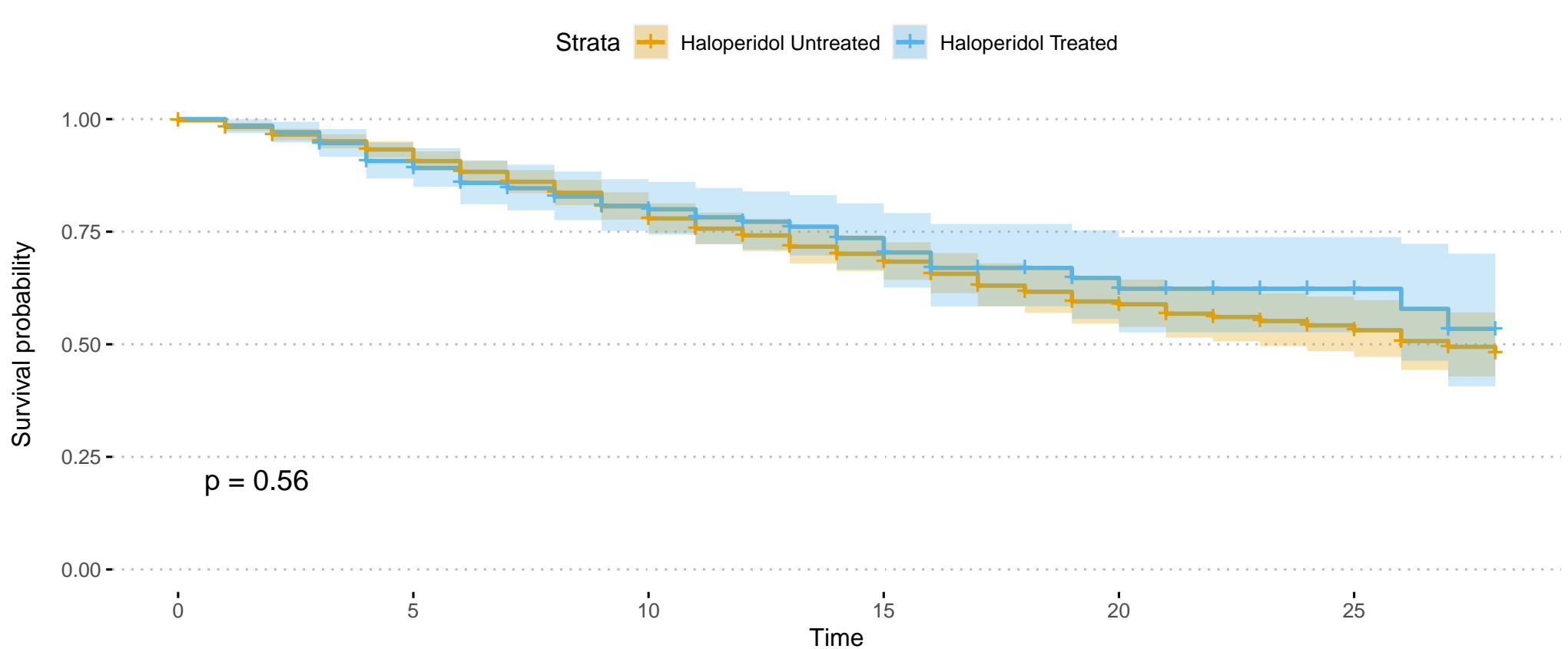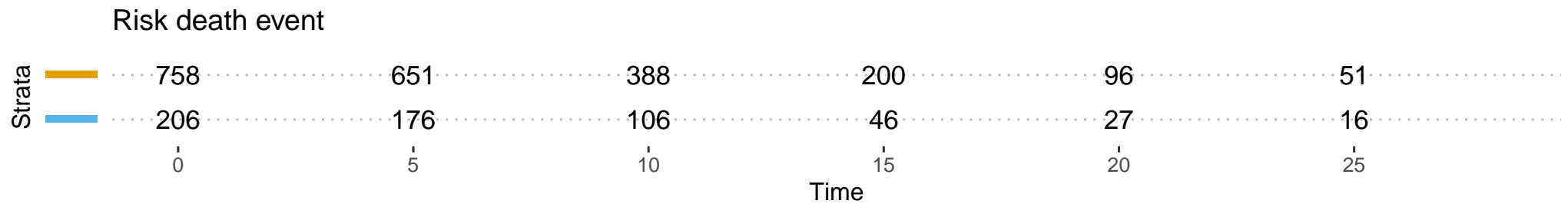

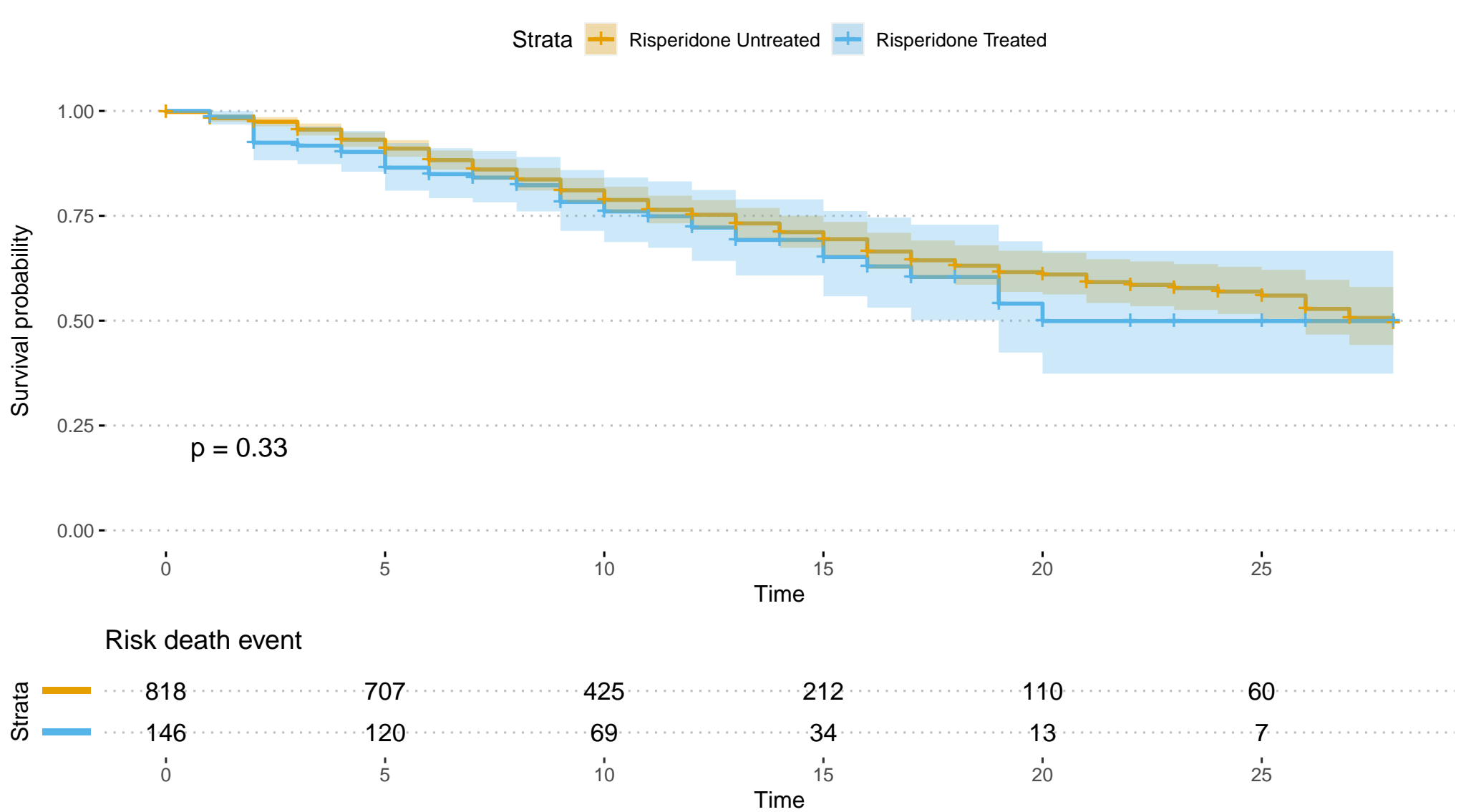

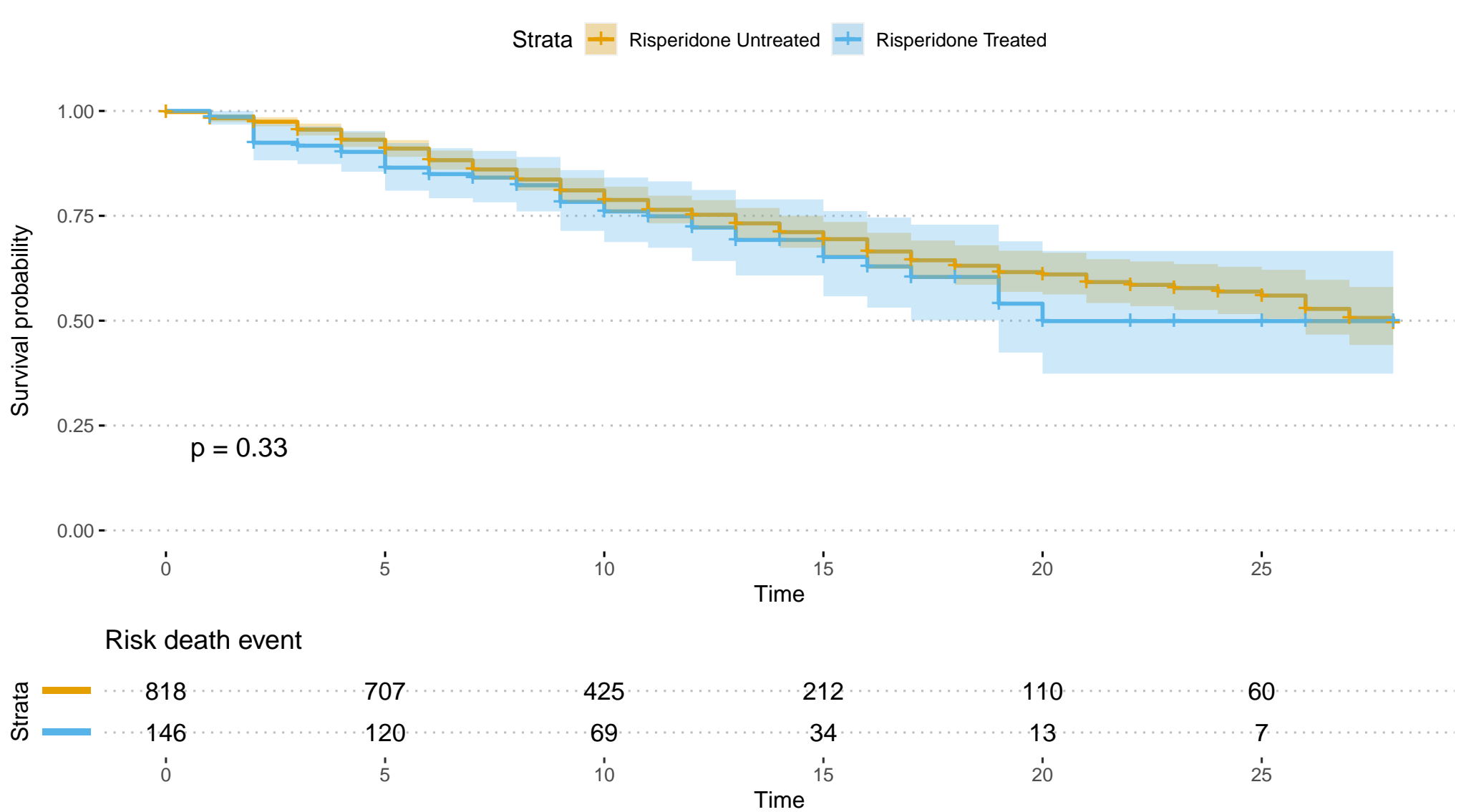

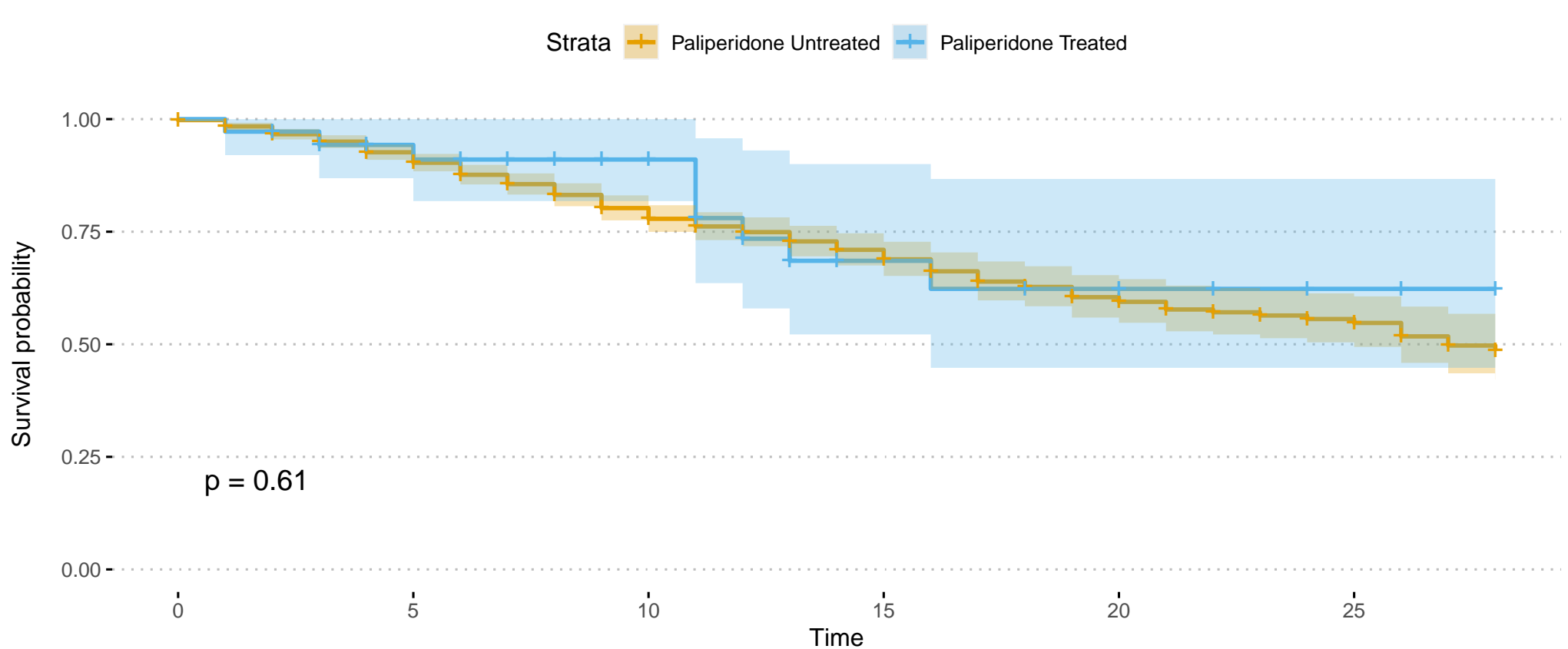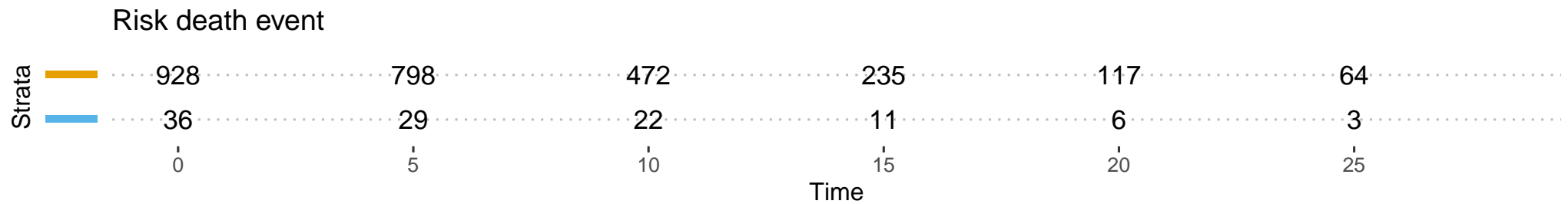

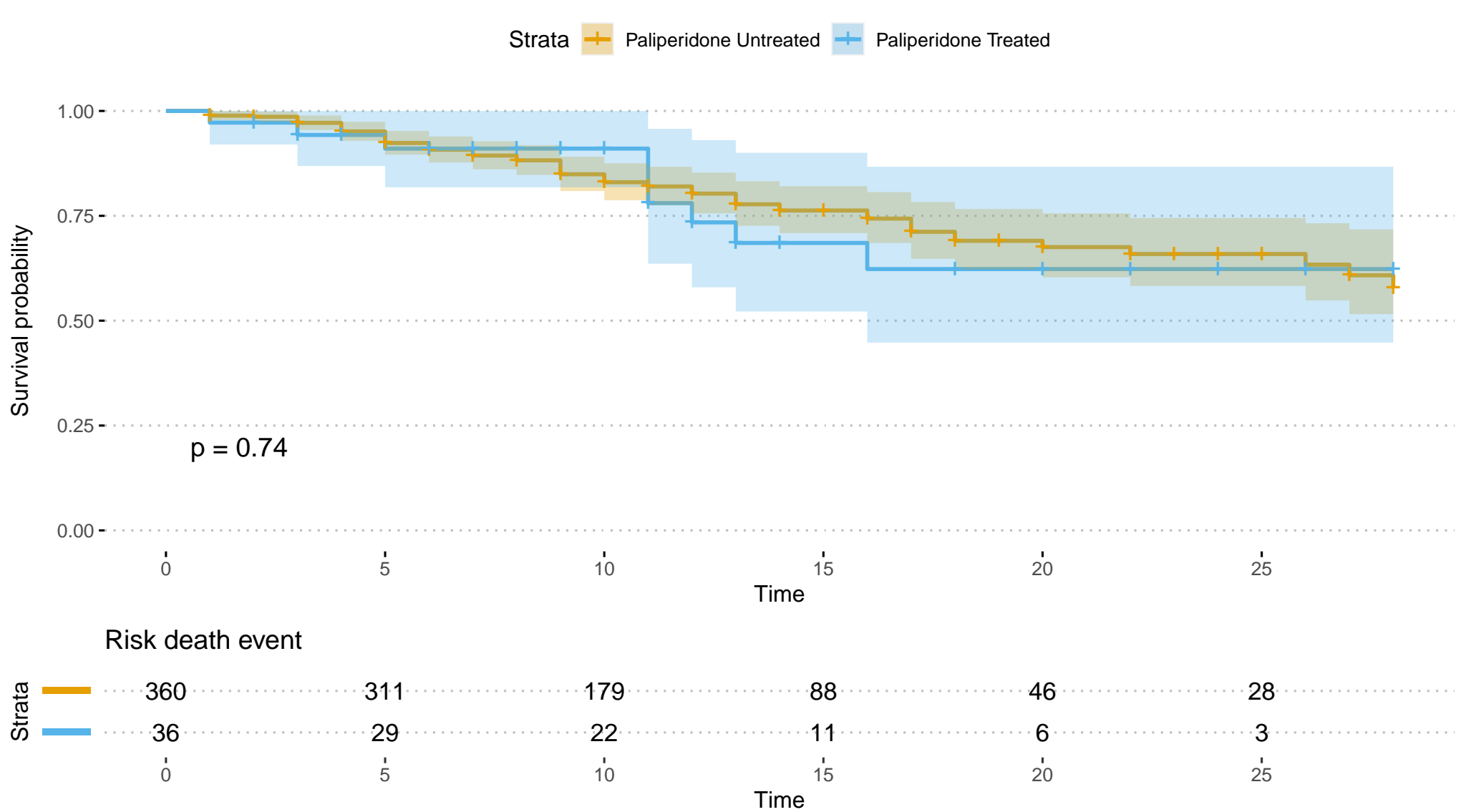

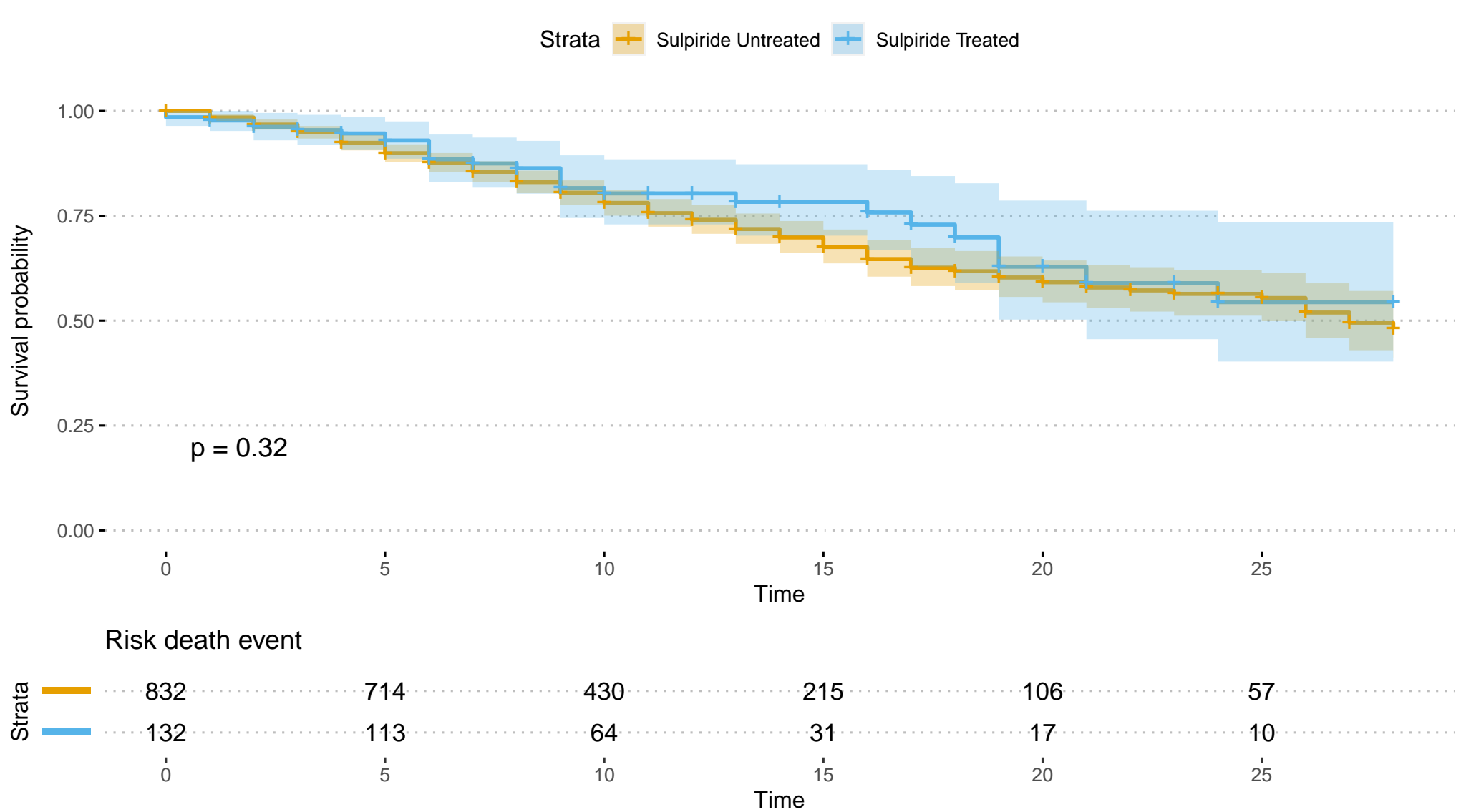

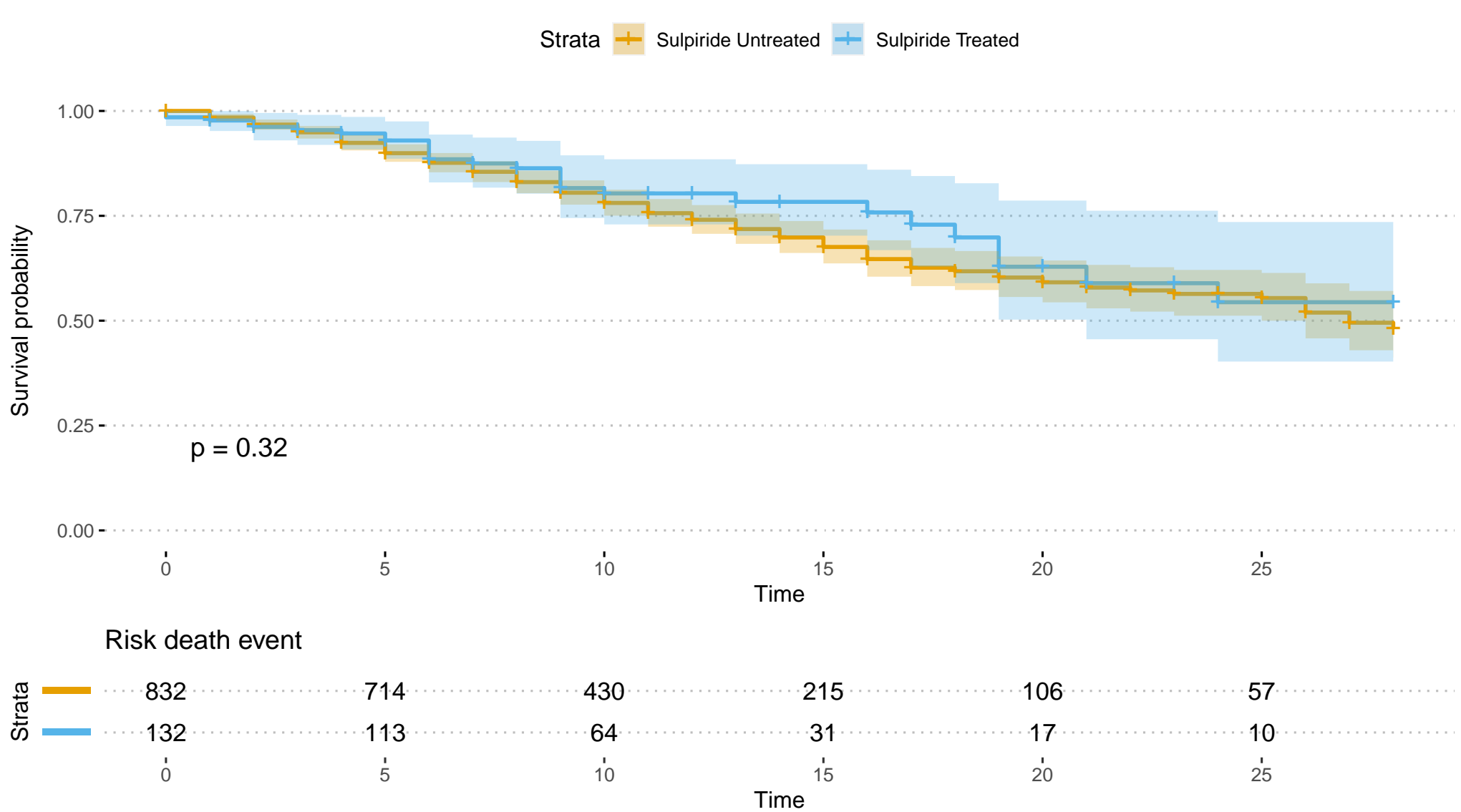

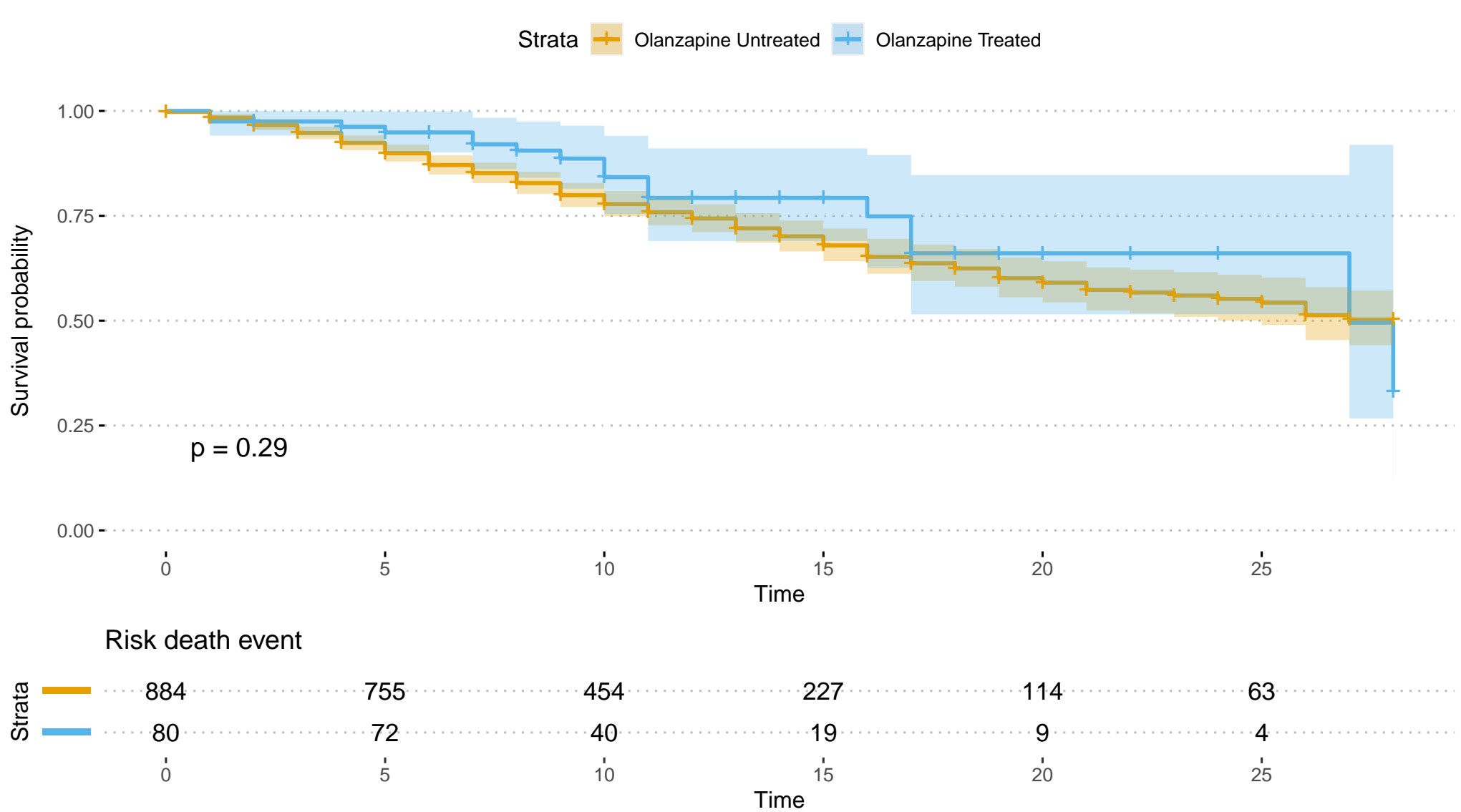

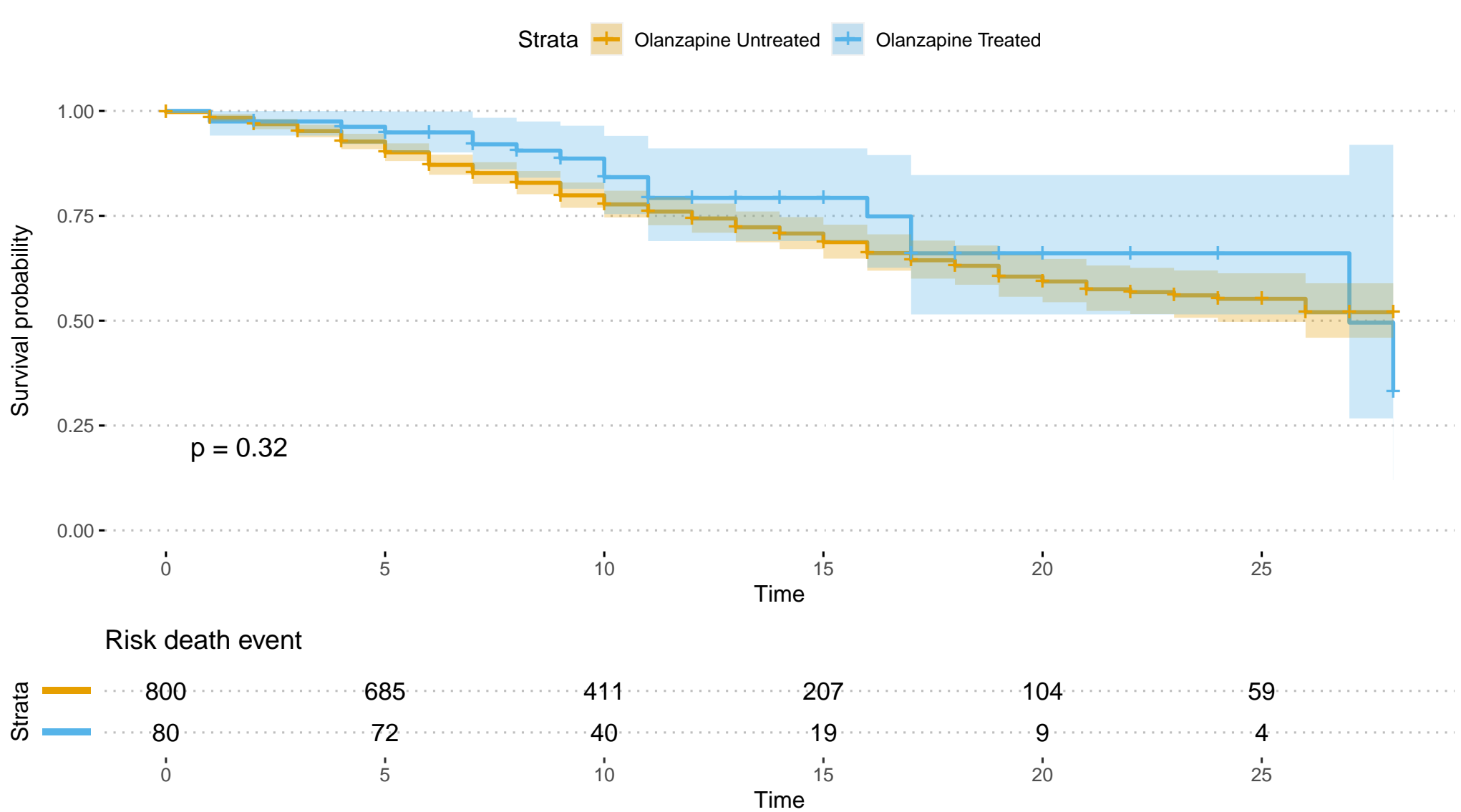

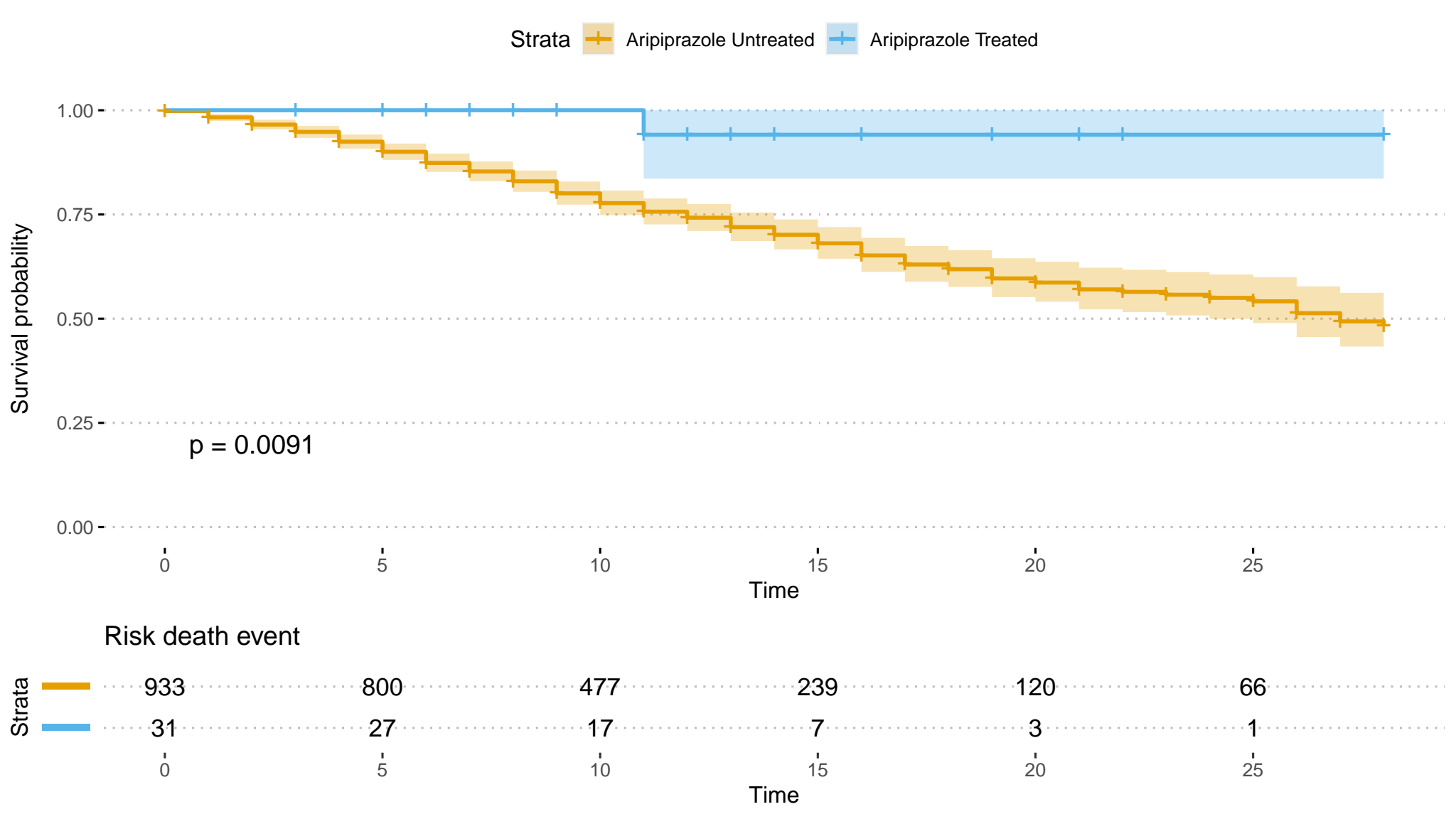

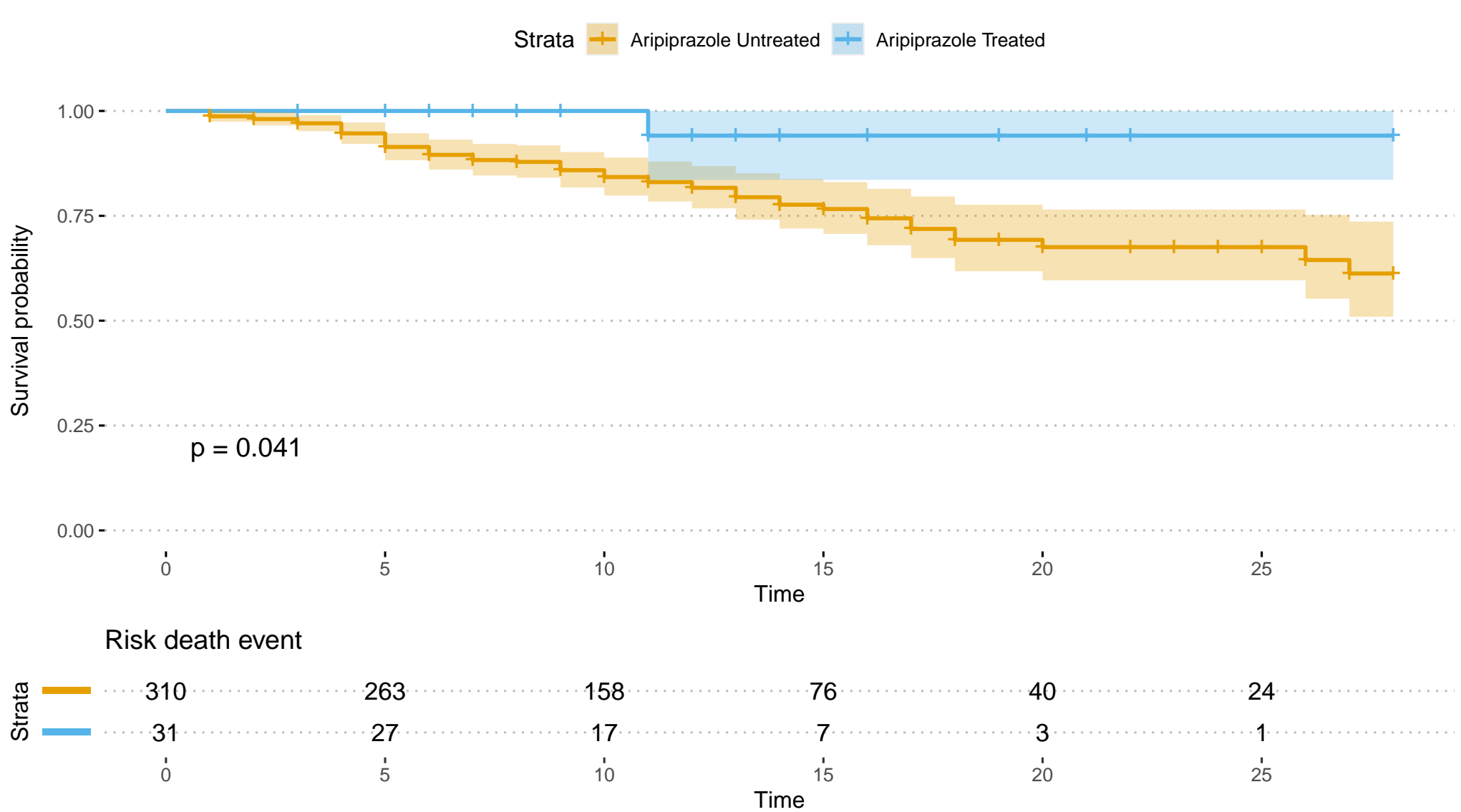

Supplement: Supplementary file 2 — Supplementary Information 2. [file 41598_2024_60297_MOESM2_ESM.pdf]
